# Toxicokinetic Studies of the Two Stimulants M-ALPHA and *N*-Methyl-cyclazodone Using In Vitro and In Vivo Tools

**DOI:** 10.3390/metabo16050291

**Published:** 2026-04-23

**Authors:** Tanja M. Gampfer, Samira Klaes, Niels Eckstein, Markus R. Meyer

**Affiliations:** 1 Department of Experimental and Clinical Toxicology, Institute of Experimental and Clinical Pharmacology and Toxicology, Center for Molecular Signaling (PZMS), PharmaScienceHub (PSH), Saarland University, 66424 Homburg, Germany; 2 Applied Pharmacy, University of Applied Sciences Kaiserslautern, Campus Pirmasens, 66953 Pirmasens, Germany

**Keywords:** synthetic stimulants, NPS, HPLC-HRMS/MS, CYP kinetic, metabolism, CYP inhibition, plasma protein binding

## Abstract

**Background/Objectives:** Synthetic stimulants represent the most prevalent subclass on the new psychoactive substances (NPSs) market. However, the toxicokinetic properties of M-ALPHA, a regioisomer of MDMA and *N*-methyl-cyclazodone a pemoline derivative, are not yet characterized. **Methods:** Therefore, this study investigated the metabolism of both NPSs in pooled liver S9 fraction and rat urine, characterized cytochrome P450 (CYP) kinetics and plasma protein binding (PPB), and assessed the CYP inhibition potential of M-ALPHA, using high-performance liquid chromatography coupled to high resolution tandem mass spectrometry (HPLC-HRMS/MS). **Results:** Four metabolites of M-ALPHA were detected including one phase I and three phase II metabolites, resulting from demethylenation followed by subsequent methylation or glucuronidation. For *N*-methyl-cyclazodone, one phase I metabolite formed via *N*-demethylation was identified. The primary enzymes involved in M-ALPHA metabolism were CYP2B6 and CYP2D6. Notably, M-ALPHA inhibited these enzymes to a strong or moderate extent, respectively. In contrast, the metabolism of *N*-methyl-cyclazodone was primarily mediated by CYP2A6. PPB studies indicated low-to-moderate binding for both compounds, suggesting that significant protein-binding interactions are unlikely. **Conclusions:** As M-ALPHA only formed metabolites that overlapped with those of MDMA, differing only by minor retention time shifts, reliable HPLC-HRMS/MS-based identification may be challenging in clinical and forensic toxicology settings as well as doping analysis. Furthermore, drug–drug interactions following polydrug use cannot be excluded for either NPS, particularly when co-ingested with other CYP substrates metabolized by the same isoforms.

## 1. Introduction

The emergence of new psychoactive substances (NPSs) on the drug abuse market remains a critical challenge for public health authorities, clinicians, and toxicological laboratories. Among the different classes of NPSs, stimulants represent the largest and most dynamic group, with nearly 400 substances recorded by the UNODC Early Warning Advisory. This category comprises several structural subgroups, including phenethylamines, cathinones, aminoindanes, and 2-amino-5-aryl-2-oxazolines [1]. The consumption of these substances is associated with severe health risks, including acute toxicity, cardiovascular complications, and long-term neurological effects [2,3,4].

**Figure 1:**
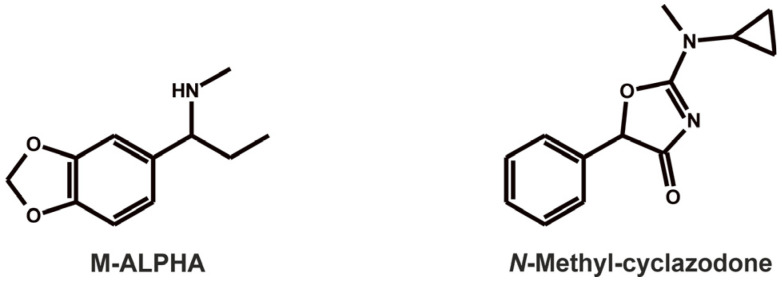
Chemical structures of the investigated stimulants.

The two NPSs investigated in this study (see Figure 1), M-ALPHA (1-methylamino-1-(3,4-methylendioxyphenyl)propane) and *N*-methyl-cyclazodone (2-[cyclopropyl(methyl)amino]-5-phenyl-1,3-oxazol-4-one) belong to this stimulant subgroup. M-ALPHA, a constitutional isomer of 3,4-methylendioxymethylamphetamin (MDMA), was first found being sold in the United Kingdom in 2010 [5]. While MDMA contains a phenethylamine backbone with the amine positioned on the β-carbon, M-ALPHA features the *N*-methylamino group at the benzylic position. Although data on its pharmacological profile are scarce, Shulgin and Shulgin (1991) reported that self-administration of a 60 mg dose produced effects similar to its *N*-demethylated homologue, ALPHA, but with roughly twice the potency and duration, resulting in a pleasant, positive mood [6]. Mechanistically, M-ALPHA is expected to act similarly to MDMA as a substrate for vesicular monoamine transporter proteins, thereby stimulating the release of serotonin, dopamine, and noradrenaline [7].

The second compound, *N*-methyl-cyclazodone, was first reported in the United States in 2022 in a presumed intoxication case. It is structurally related to pemoline and cyclazodone, both containing an oxazolone moiety. Pemoline, a presynaptic releaser and reuptake blocker of dopamine, was formerly used in the treatment of attention-deficit hyperactivity disorder and has also been investigated in clinical studies as a potential therapeutic candidate for narcolepsy [8,9]. However, it was withdrawn from the market due to rare but idiosyncratic hepatotoxicity [10]. Its closest structural analogue, cyclazodone, was developed in the 1960s, but despite showing strong stimulant and anorexigenic effects, it was never approved for therapeutic use [11]. 

While evidence from structurally related compounds points towards a similar pharmacological and toxicological profile of M-ALPHA and *N*-methyl-cyclazodone, no empirical data currently exist regarding the toxicokinetics of M-ALPHA or *N*-methyl-cyclazodone. To address this knowledge gap, the present study characterized the in vitro and in vivo toxicokinetic properties of the two stimulants using high-performance liquid chromatography coupled to high-resolution tandem mass spectrometry (HPLC-HRMS/MS). This included the identification of metabolites in pooled human liver S9 fraction (pHLS9) and rat urine together with detectability studies using standard urine screening approaches (SUSA), elucidation of cytochrome P450 (CYP) enzyme kinetic profiles, assessment of the CYP enzyme inhibition potential of M-ALPHA, and determination of plasma protein binding (PPB).

## 2. Materials and Methods

### 2.1. Chemicals and Enzymes

M-ALPHA hydrochloride (HCl) and *N*-methyl-cyclazodone were provided by University of Applied Sciences Kaiserslautern from a meanwhile closed online shop for NPSs (Germany). Verapamil HCl were obtained from Sigma-Aldrich (Steinheim, Germany), bupropion HCl from GlaxoSmithKline (Munich, Germany), dextromethorphan hydrobromide from Roche (Grenzach, Germany), quinidine sulfate from Chininfabrik Buchler (Braunschweig, Germany) sertraline from Pfizer (Berlin, Germany), and testosterone from Fluka (Neu-Ulm, Germany). S-(5′-Adenosyl)-l-methionine (SAM), dithiothreitol (DTT), isocitrate, isocitrate dehydrogenase, magnesium chloride (MgCl_2_), 3′-Phosphoadenosine-5′-phosphosulfate (PAPS), reduced glutathione (GSH), superoxide dismutase, and trimipramine-d3 (internal standard, IS) were supplied by Merck (Taufkirchen, Germany). Centrifree devices were provided by Merck (Darmstadt, Germany), diazepam-d5 was from Lipomed (Weil am Rhein, Germany), and acetyl coenzyme A (AcCoA), dipotassium hydrogen phosphate (K_2_HPO_4_), potassium dihydrogen phosphate (KH_2_PO_4_), and Tris were obtained from Sigma-Aldrich (Taufkirchen, Germany). Nicotinamide adenine dinucleotide phosphate (NADP^+^) was from Biomol (Hamburg, Germany). Acetonitrile (HPLC-MS grade), methanol (HPLC-MS grade), ammonium formate (analytical grade), formic acid (HPLC-MS grade), and all other reagents and chemicals (analytical grade) were supplied by VWR International (Darmstadt, Germany). The baculovirus-infected insect cell microsomes (ICM) containing the human complementary cDNA expressed CYP enzymes CYP1A2, CYP2B6, CYP2C8, CYP2C19, CYP2D6, CYP3A4, CYP3A5 (1 nmol/mL, each), CYP2A6, CYP2C9, CYP2E1 (2 nmol/mL, each), flavin-containing monooxygenase (FMO3, 5 mg protein/mL), pooled human liver microsomes (pHLM, 20 mg microsomal protein/mL, 240 pmol total CYP/mg protein, 35 individual donors), pHLS9 (20 mg microsomal protein/mL, 30 individual donors), uridine 5′-diphospho-glucuronosyltransferase (UGT) reaction mixture solution A (25 mM uridine 5′-diphosphoglucuronic acid) and UGT reaction mixture solution B (250 mM Tris HCl, 40 mM MgCl_2_, and 125 µg alamethicin/mL) were supplied by Discovery Life Sciences (Huntsville, AL, USA). After delivery, the enzyme preparations were thawed at 37 °C, aliquoted, snap-frozen in liquid nitrogen, and stored at −80 °C until use.

### 2.2. pHLS9 Incubations

As described elsewhere [12], the final incubations contained 2 mg protein/mL and the final volume was 300 µL. All given concentrations are final concentrations in the incubation mixtures. First, a mixture of 0.1 mM AcCoA, 25 µg/mL alamethicin (UGT reaction mixture solution B), 2.5 mM isocitrate, 0.8 U/mL isocitrate dehydrogenase, 2.5 mM MgCl_2_, 0.6 mM NADP+, 2 mg protein/mL pHLS9, 90 mM phosphate buffer (pH 7.4), and 100 U/mL superoxide dismutase was preincubated at 37 °C for 10 min. Afterwards, 1 mM DTT, 10 mM GSH, 40 mM PAPS, 1.2 mM SAM, and 2.5 mM uridine 5′-diphosphoglucuronic acid (UGT reaction mixture solution A), were added. Reactions were started by the addition of 25 µM M-ALPHA or *N*-methyl-cyclazodone, respectively. Reaction mixtures were incubated at 37 °C for 360 min. A volume of 30 µL was transferred to a new reaction tube after 60 and 360 min of incubation. Thereafter, 10 µL of ice-cold acetonitrile (+2.5 µM trimipramine-d3) were added to stop reactions. Afterwards, sample tubes were cooled at −20 °C for 30 min and centrifuged at 18.07× *g* for 2 min. Finally, the supernatants (20 µL) were transferred into autosampler vials for HPLC-HRMS/MS analysis. Blank incubations without substrate and control incubations without pHLS9 were also performed to identify interfering compounds and compounds of non-metabolic origin. All incubations were performed twice.

### 2.3. Rat Urine Collection and Sample Preparation

Animal experiments were conducted in accordance with German legislation, including the Animal Welfare Act and EU Directive 2010/63/EU, and were approved by an ethics committee (Landesamt für Verbraucherschutz, Saarbrücken, Germany, 33/2019). A single oral low dose of 2 mg/kg stimulants were administered to the male Wistar rats (Charles River, Sulzfeld, Germany). Rats had water ad libitum during the collection of urine over a period of 24 h. Before compound administration, blank urine was collected to confirm the absence of interfering compounds.

Sample preparation was based on a published procedure [13] with minor modifications. Briefly, 100 µL of rat urine was precipitated with 500 µL of ice-cold acetonitrile containing 0.7 µM diazepam-d5 as IS. The samples were mixed for 2 min and centrifuged at 18,407× *g* for 2 min. The resulting supernatants were evaporated to dryness at 70 °C under a nitrogen stream and reconstituted in 50 µL of a 50:50 (*v*/*v*) mixture of aqueous 10 mM ammonium formate containing 0.1% formic acid (*v*/*v*) and acetonitrile containing 0.1% formic acid (*v*/*v*). The samples were subsequently analyzed under the HPLC-HRMS/MS conditions described below.

### 2.4. Standard Urine Screening Approaches for Detectability Studies

The SUSA followed published procedures: GC–MS SUSA [14,15], HPLC-LRMSn SUSA [16,17], and HPLC-HRMS/MS SUSA [18,19].

### 2.5. Screening for CYP Enzyme Contribution

Incubations were carried out according to a standard protocol [20]. All given concentrations are final concentrations in the incubation mixtures. Incubations contained 25 μM M-ALPHA or *N*-methyl-cyclazodone and 50 pmol/mL of CYP1A2, CYP2A6, CYP2B6, CYP2C8, CYP2C9, CYP2C19, CYP2D6, CYP2E1, CYP3A4, CYP3A5, or 0.25 mg/mL of FMO3, respectively. Additionally, the incubation mixtures consisted of 5 mM isocitrate, 0.5 U/mL isocitrate dehydrogenase, 5 mM MgCl_2_, 1.2 mM NADP+, 90 mM phosphate buffer, and 200 U/mL superoxide dismutase. Incubations with CYP2A6 and CYP2C9 were performed with Tris buffer instead of phosphate buffer as recommended by the manufacturer’s manual. Reactions were started by the addition of a prewarmed mixture (37 °C) of isocitrate, isocitrate dehydrogenase, MgCl_2_, and NADP^+^. Incubations were performed for 30 min at 37 °C (final volume: 100 µL). Afterwards, 50 µL were transferred to a new reaction tube containing 50 µL of ice-cold acetonitrile (+2.5 µM trimipramine-d3) to stop reactions, followed by centrifugation at 18,407× *g* for 5 min. Lastly, supernatants were transferred to autosampler vials for HPLC-HRMS/MS analysis as described under 2.9. Positive control incubations with pHLM (1 mg protein/mL) were also conducted. Blank incubations without substrate and control incubations without pHLS9 were also performed to identify interfering compounds and compounds of non-metabolic origin. All incubations were performed twice.

### 2.6. Kinetic Studies

Based on prior publications [21,22], M-ALPHA was incubated with CYP2D6, CYP2C19, and pHLM for 10 min and with CYP2B6 and CYP3A4 for 5 min, respectively. Incubations of *N*-methyl-cyclazodone with CYP2A6 and pHLM were conducted for 15 min each, with CYP2C19 for 20 min and with CYP1A2 for 5 min. All reported concentrations refer to final concentrations in the incubation mixtures. Incubation times and CYP enzyme concentrations were selected to fall within the linear range of metabolite formation, as determined from preliminary incubations of the stimulants. These variables were evaluated by plotting the peak area ratio (PAR) of the metabolite to the IS against protein concentration or incubation time, and the linear range was visually determined from the resulting plots. Protein concentrations for M-ALPHA incubations were 20–50 pmol/mL for the CYP isozymes and 0.06 mg/mL for pHLM. For the *N*-methyl-cyclazodone experiment, the CYP enzyme concentrations ranged from 20 to 30 pmol/mL, while pHLM was used at 0.06 mg/mL. Substrate concentrations for both compounds ranged from 1 to 1000 µM, except for the CYP2D6 incubation of M-ALPHA (0.1–2 µM) and for CYP2A6 of *N*-methyl-cyclazodone (1–500 µM). All substrate concentrations were incubated in duplicate, along with a blank control containing either Tris or phosphate buffer in place of enzymes to identify interfering compounds and compounds of non-metabolic origin. All other incubation conditions were consistent as aforementioned for microsomal incubations. The final incubation volume was 100 µL. Reactions were terminated by transferring 25 µL of the incubation mixture into a new reaction tube containing 25 µL ice-cold acetonitrile. Samples were centrifuged at 18,407× *g* for 2 min. An aliquot of 20 µL of the supernatant was transferred to an autosampler vial and analysis was performed using HPLC-HRMS/MS as described below.

The PAR of the metabolite and IS were used to estimate the relative amount of metabolite formed. Based on these values, the relative in vivo hepatic net clearance was calculated. Enzyme kinetic constants were determined by nonlinear regression using GraphPad Prism 5.00 software (GraphPad Software, San Diego, CA, USA). The Michaelis–Menten equation (Equation (1)) was used to calculate the apparent Michalis-Menten constant (K_m_) and maximum reaction velocity (V_max_) based on the substrate concentration [S] for individual CYP enzymes.

(1) $$V=\frac{V_{max}\times \left[S\right]}{K_{m}+\left[S\right]}$$

In addition, the relative activity factor (RAF) was calculated (Equation (2)) to account for differences in the functional levels of redox partners between the two enzyme sources [23]. Turnover rates (TR) of the CYP enzymes in pHLM and ICM were taken from the suppliers’ data sheets.

(2) $${RAF}_{enzyme}=\frac{TR\;in\;pHLM}{TR\;in\;ICM}$$

The enzyme velocities (V_enzyme_) were calculated at two different substrate concentrations (0.1 and 10 µM) and subsequently multiplied by the corresponding RAF. This yielded a value defined as contribution_enzyme_ (Equation (3)). The V_max_ and K_m_ values (Equation (1)) were derived from the incubations using isolated CYP enzymes.

(3) $${contribution}_{enzyme}={RAF}_{enzyme}\times V_{enzyme}\;$$

Equation (4) was used to calculate the percentage of net clearance by a specific CYP enzyme at a given substrate concentration.

(4) $${clearance}_{enzyme}\left[\%\right]=\frac{{contribution}_{enzyme}}{{\sum contribution}_{enzyme}}\times 100$$

### 2.7. CYP Inhibition Assay of M-ALPHA

The CYP inhibition study followed published protocols, with certain modifications [24,25]. Instead of using a dual-cocktail approach, M-ALPHA was incubated separately with the individual substrates and inhibitors of each metabolically relevant CYP enzyme. Bupropion was used as the model substrate of CYP2B6 and formation of 4-hydroxybupropion was monitored. The model substrate of CYP2D6 was dextromethorphan, with O-demethyl dextromethorphan recorded as the corresponding metabolite. For CYP3A4/5, testosterone was used as selective substrate and 6-β-hydroxytestosterone was monitored as metabolite. Inhibitors specific to each CYP enzyme were as follows: sertraline (CYP2B6), quinidine (CYP2D6), and verapamil (CYP3A4/5).

All stock solutions were freshly prepared from the pure substance. Bupropion, dextromethorphan, sertraline, and verapamil (1 mg/mL) were prepared in water, whereas the quinidine stock solution (1 mg/mL) was prepared in methanol/water (50:50, *v*/*v*). The testosterone stock solution (1 mg/mL) was prepared in a methanol/water mixture (33:66, *v*/*v*). The composition of the reaction mixture was the same as described in Section 2.5, with the addition of CYP-specific substrates at final concentrations of 86 µM testosterone, 8.9 µM dextromethorphan, and 30 µM bupropion, corresponding to the CYP enzyme tested. M-ALPHA was used at 100 µM. Reactions were initiated by adding the respective microsomal enzyme (final volume: 50 µL) and terminated after 15 min by the addition of 50 µL ice-cold acetonitrile (+2.5 µM trimipramine-d3). Afterwards, samples were centrifuged at 18,407× *g* for 5 min and the supernatants were transferred to autosampler vials for analysis by LC-HRMS/MS as described in Section 2.8.

Additionally, negative controls and positive controls were prepared. The positive control contained the respective model inhibitor at 20 µM. The negative control, containing buffer instead of inhibitor, was intended to confirm metabolite formation. All incubations were performed in triplicate. Evaluation of CYP inhibition by M-ALPHA was done by monitoring the peak area of the respective metabolite. The peak area in the negative control was set to 100%. The remaining CYP enzyme activity, expressed as percentage, was then calculated by comparing the peak areas of the respective metabolite in the test samples and positive controls to those in the negative control.

### 2.8. Plasma Protein Binding Studies

The PPB study was done in accordance with published procedures [12,24]. A volume of 50 μL of methanolic M-ALPHA and *N*-methyl-cyclazodone solution (final concentration 3 μM) was spiked into 450 μL of fresh pooled human plasma and incubated for 30 min at 37 °C. Thereafter, a 100 μL sample (global approach, GA) was taken and transferred into a new reaction tube. The remaining sample was transferred into an ultrafiltration device and centrifuged at 1600× *g* for 35 min. A volume of 100 μL of the ultrafiltrate (UF) was transferred into a new reaction tube. All samples were precipitated by adding 50 μL ice-cold acetonitrile containing trimipramine-d3 (2.5 μM) as IS and 0.1% (*v*/*v*) formic acid, cooled for 30 min at −20 °C and centrifuged for 2 min at 18,407× *g* before measurement. Ultrafiltration was done in triplicate, respectively. Based on the unbound fraction in plasma (*f*_u_) the PPB was calculated using the following equations:

(5) $$f_{u}=\frac{PAR\left(\frac{{Stimulant}_{UF}}{{IS}_{UF}}\right)}{PAR\left(\frac{{Stimulant}_{GA}}{{IS}_{GA}}\right)}$$

(6) $$PPB,\;\%=\left(1-f_{u}\right)\times 100$$

ChemDraw (Revvity Signals, Waltham, MA, USA; v. 25.5.5789) was used to calculate the compounds’ hydrophilic properties (cLog P).

### 2.9. HPLC-HRMS/MS Parameters

Mass spectrometric analysis was performed using a Q-Exactive Plus mass spectrometer (ThermoFisher Scientific, TF, Dreieich, Germany) with a heated electrospray ionization (HESI)-II source. This system was further coupled to a TF Dionex UltiMate 3000 pump comprising a degasser, a quaternary pump, and an UltiMate Autosampler. Chromatographic conditions followed the procedure outlined by Kroesen et al. [20]. Chromatographic separation was achieved using a TF Accucore Phenyl-Hexyl column (100 mm × 2.1 mm × 2.6 μm). Eluent A consisted of 2 mM aqueous ammonium formate containing 0.1% formic acid (*v*/*v*), while Eluent B comprised 2 mM ammonium formate with a methanol:acetonitrile mixture (50:50, 1%, *v*/*v*), water (1%, *v*/*v*), and formic acid (0.1%, *v*/*v*). The flow rate was maintained at 0.5 mL/min from 0 to 10 min and increased to 0.8 mL/min from 10 to 13.5 min. The gradient was programmed as follows: 0–1 min, hold at 99% eluent A; 1–10 min, decreased to 1% A; 10–11.5 min, hold at 1% A; 11.5–13.5 min, re-equilibration at 99% A. The injection volume was 5 μL for all samples. The HESI-II source was operated under the following conditions: heater temperature, 320 °C; ion transfer capillary temperature, 320 °C; spray voltage, 4.0 kV; ionization mode, positive; sheath gas, 60 arbitrary units (AU); auxiliary gas, 10 AU; sweep gas, 0 AU; S-lens RF level, 60.0. Mass spectrometric analysis was performed in full scan (FS) mode and subsequent data-dependent acquisition (DDA) prioritizing mass-to-charge ratios (*m*/*z*) of the parent compounds M-ALPHA and *N*-methyl-cyclazodone along with their expected metabolites. The following settings for FS data acquisition were used: resolution, 35,000 FWHM at *m*/*z* 200; microscans, 1; automatic gain control (AGC) target, 1E6; maximum injection time (maxIT), 120 ms; scan range, *m*/*z* 80–900. The following DDA mode settings were used: option “pick others”, enabled; dynamic exclusion, feature not used; resolution, 17,500 FWHM at *m*/*z* 200; microscans, 1. ChemSketch (ACD/Labs, Toronto, ON, Canada; v. 2023.1.2) was used to draw chemical structures of both stimulants together with their expected phase I and II metabolites and to calculate the exact masses. One method was developed for both compounds with one inclusion list including all predicted metabolites. The collected data were analyzed using TF Xcalibur Qual Browser software (v. 4.6).

## 3. Results and Discussion

### 3.1. Metabolite Identification

**Figure 2:**
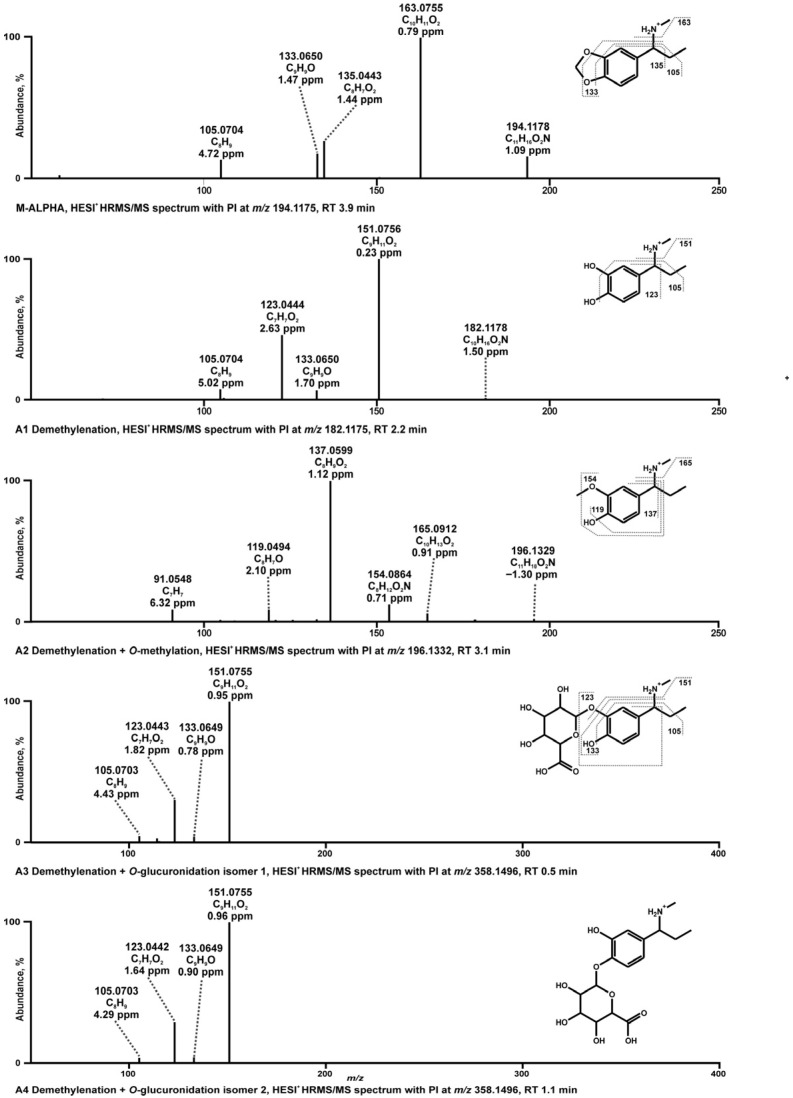
HRMS/MS spectra of proposed in vitro and in vivo phase I and II metabolites of M-ALPHA.

**Figure 3:**
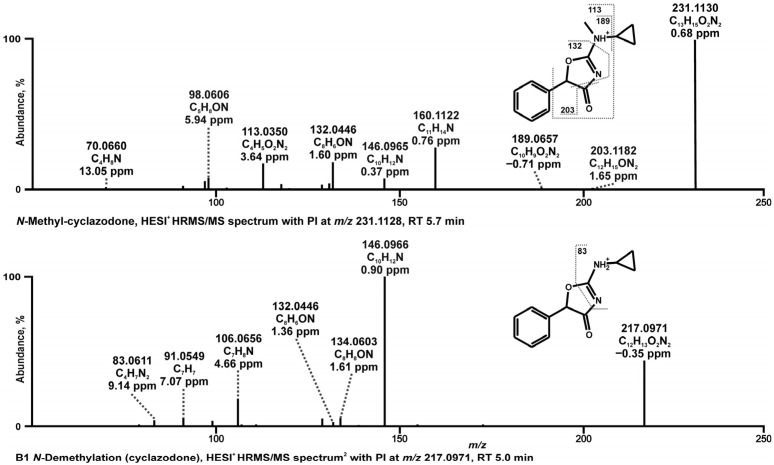
HRMS/MS spectra of proposed in vitro and in vivo phase I metabolites of *N*-methyl-cyclazodone.

Metabolites were tentatively identified by comparing the HRMS/MS of the parent compound to those of the metabolites, based on the interpretation of precursor ions (PIs) and fragmentation ion (FI) patterns. The corresponding spectra are shown in Figure 2 and Figure 3 and further details are available in Tables S1 and S2 (Supporting Information). The exact (calculated) masses of parent compounds and their metabolites are used in the following section for discussion.

#### 3.1.1. Tentative Metabolites and Proposed Fragmentation Patterns of M-ALPHA

The HRMS/MS spectrum of M-ALPHA (C_11_H_15_O_2_N), with PI at *m*/*z* 194.1175, contained an FI at *m*/*z* 163.0754 (C_10_H_11_O_2_), corresponding to the loss of a methylamine group. Further cleavage of the methylene bridge within the methylenedioxy ring resulted in an FI at *m*/*z* 151.0753 (C_9_H_11_O_2_). From this FI, the loss of a terminal methyl group yielded an FI at *m*/*z* 135.0440 (C8H7O2). Subsequent loss of the two catechol hydroxyl groups produced FI at *m*/*z* 105.0699 (C_8_H_9_).

One phase I metabolite and three phase II metabolites were identified. The phase I metabolite (A1) with a precursor ion (PI) at *m*/*z* 182.1175 (C_10_H_16_O_2_N) resulted from the demethylenation of the methylenedioxy ring. The HRMS/MS spectrum of A1 showed similarities to the parent compound, as indicated by shared FIs at *m*/*z* 151.0753, 135.0440, and 105.0699, although with differing relative intensities. Notably, an FI at *m*/*z* 123.0443 (C_7_H_7_O_2_) was observed exclusively in the spectrum of demethylenated M-ALPHA, likely originating from the FI at *m*/*z* 151.0753 via loss of an ethylene group. The absence of an FI at *m*/*z* 123.0443 in the parent spectrum might be due to the low abundance of the FI at *m*/*z* 151.0753. The absence of FI at *m*/*z* 163.0754 in the metabolite spectrum, indicative of the intact methylenedioxy ring, further supports this metabolic reaction.

The first phase II metabolite (A2) with PI at *m*/*z* 196.1332 (C_11_H_18_O_2_N) was formed by methylation of metabolite A1 via catecholamine-*O*-methyltransferases. The FI at *m*/*z* 165.0912 corresponded to the parent FI at *m*/*z* 163.0755 shifted by two mass units due to the structural modification by two hydrogens. The second and third phase II metabolite (A3 and A4) were isomers generated by demethylenation followed by *O*-glucuronidation at one of the aromatic hydroxy groups with PI at *m*/*z* 358.1496, respectively. A reliable identification was possible as fragmentation patterns were similar to the phase I metabolite A1, although the PIs were not detectable. It should be noted that the exact position of the glucuronides at one of the hydroxy groups could not be determined and was assigned randomly.

#### 3.1.2. Tentative Metabolites and Proposed Fragmentation Pattern of *N*-Methyl-cyclazodone

**Figure 4:**
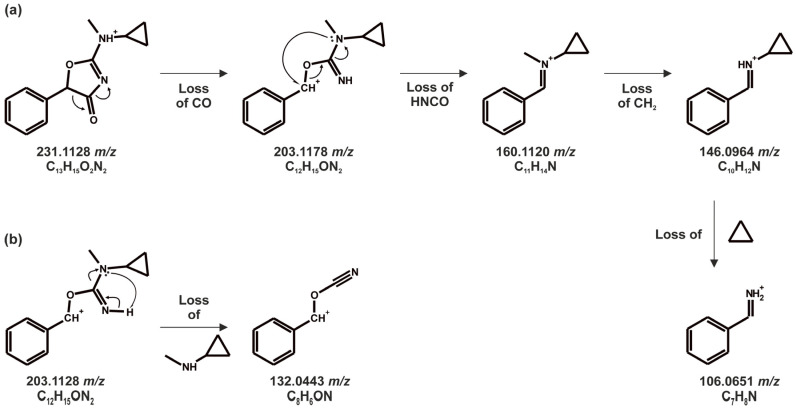
Proposed HRMS/MS rearrangement and fragmentation mechanism of *N*-methyl-cyclazodone. (**a**) Ring opening mechanism with rearrangement via loss of isocyanic acid. (**b**) Rearrangement mechanism after ring opening via loss of *N*-cyclopropylamine.

*N*-Methyl-cyclazodone (C_13_H_14_O_2_N_2_), with a PI at *m*/*z* 231.1128, showed a minor abundant FI at *m*/*z* 189.0660 (C_10_H_9_O_2_N_2_), formed by cleavage of the cyclopropyl group. Subsequent phenyl ring cleavage resulted in the formation of FI at *m*/*z* 113.0345 (C_4_H_5_O_2_N_2_). However, several FIs, including *m*/*z* 203.1178 (C_12_H_15_ON_2_), 160.1120 (C_11_H_11_N), and 146.0964 (C_10_H_11_N), could not be assigned to this fragmentation pathway, suggesting a rearrangement of the 4-amino-oxazolone moiety. To our knowledge, such rearrangements for 4-amino-oxazolone-containing compounds (as proposed in Figure 4a) have not been previously reported. In the proposed mechanism (Figure 4a), the initial loss of CO (−27.9949 u) from the PI led to the formation of an FI at *m*/*z* 203.1178 (C_12_H_15_O_2_N), consistent with oxazolone ring opening. We propose that the FI at *m*/*z* 160.1120 (C_11_H_14_N) was formed via nucleophilic attack by the nitrogen next to the cyclopropyl group at the benzylic carbenium, followed by cleavage of the isocyanic acid (-HNCO) moiety. Subsequent loss of the methyl group from the nitrogen yielded the FI at *m*/*z* 146.0964 (C_10_H_12_N). Additionally, loss of the *N*-cyclopropylamine group from the FI at *m*/*z* 203.1178 (C_13_H_14_O_2_N_2_) led to the FI at *m*/*z* 132.0443 (C_8_H_6_ON) (Figure 4b).

Only one phase I metabolite and no phase II metabolites were identified for *N*-methyl-cyclazodone. This metabolite (B1, C_12_H_13_O_2_N_2_), observed with PI at *m*/*z* 217.0971, resulted from *N*-demethylation from cyclazodone. The base peak FI at *m*/*z* 146.0964 corresponded to that observed in the parent spectrum. Subsequent cleavage of the cyclopropyl group from this FI yielded the FI at *m*/*z* 106.0651 (C_7_H_8_N).

#### 3.1.3. Toxicological Detection by Standard Urine Screening Approaches

The dose used for detectability studies was at 2 mg/kg. Application of the dose-by-factor approach allows for the calculation of the human-equivalent dose from the rat dose [26]. The rat dose of 2 mg/kg body weight results in a human-equivalent dose of about 20 mg if a body weight of 60 kg was assumed. No human doses for the studied compounds were published to far, but 37.5 to 50 mg were described as single doses of pemoline [27]. Therefore, single doses of *N*-methyl-cyclazodone might be roughly twice as high, with even higher doses occurring in cases of intoxication.

Possibly due to these low levels, neither the parent compounds nor their metabolites were detectable via the GC-MS SUSA. Using the HPLC-LRMSn SUSA, M-ALPHA was detected along with the demethylenyl metabolite A1 and the two glucuronide isomers A3 and A4. In case of *N*-methyl cyclazodone, only its *N*-demethyl metabolite B1 was found by HPLC-LRMSn. Using HPLC-HRMS/MS SUSA, M-ALPHA and its demethylenyl metabolite A1 were detected, while for *N*-methyl cyclazodone only its metabolite B1 (cyclazodone) was identified. The lack of the detection of both M-ALPHA glucuronides via HPLC-HRMS/MS SUSA might be explained by the shorter gradient and a more rapid increase in polar eluent compared to HPLC-LRMSn SUSA leading to an early retention time of <1 min.

#### 3.1.4. In Vitro and In Vivo Metabolism

In vitro assays were conducted using pHLS9 to allow an initial rapid assessment of metabolism. To complement the in vitro data, rat urine was analyzed to provide a more comprehensive evaluation of in vivo metabolism. In pHLS9, only the metabolite resulting from demethylenation was detected for M-ALPHA and no phase II metabolites were observed, most likely due to their low concentrations. In rat urine, all the above-described M-ALPHA metabolites including the demethylenyl metabolite and three phase II metabolites were identified. While the demethylenation of MDMA has been associated with cardiotoxicity [28], the toxicological relevance of this pathway for the corresponding M-ALPHA metabolite remains to be elucidated. Unlike MDMA [29,30], M-ALPHA did not undergo *N*-demethylation either in vitro or in vivo models. This is likely due to steric hindrance as the *N*-methylamino group in M-ALPHA is positioned more closely to the bulky methylenedioxy moiety compared to MDMA.

Overall, all M-ALPHA metabolites described above are also known metabolites of MDMA [30]. Furthermore, both M-ALPHA and MDMA, and their shared metabolites, display nearly identical mass spectra, differing only by minor retention time shifts. Thus, differentiation between these two regioisomers in human biosamples remains challenging.

*N*-Methyl-cyclazodone was only metabolized to cyclazodone both in vitro and in vivo, possibly due to its already hydrophilic character. Urinary excretion of unchanged parent compound was therefore observed in rat urine, consistent with observations reported for pemoline after human administration [31].

### 3.2. CYP-Mediated Metabolism

The involvement of the ten most prevalent drug-metabolizing CYP enzymes CYP1A2, CYP2A6, CYP2B6, CYP2C8, CYP2C9, CYP2C19, CYP2D6, CYP2E1, CYP3A4, and CYP3A5 together with FMO3 was assessed in the metabolism of M-ALPHA and *N*-methyl-cyclazodone. Demethylenation of M-ALPHA was primarily mediated by CYP2B6, CYP2C19, CYP2D6, and CYP3A4. This observation is consistent with published data on MDMA metabolism, although CYP1A2 was not involved in the demethylenation reaction, likely due to steric effects [29]. In contrast, CYP1A2, CYP2A6, and CYP2C19 contributed to the formation of cyclazodone. The role of CYP enzymes in the metabolism of closely related *N*-methyl-cyclazodone analogues has not yet been reported.

### 3.3. CYP Kinetic Studies and Calculation of In Vivo Hepatic Net Clearance

**Figure 5:**
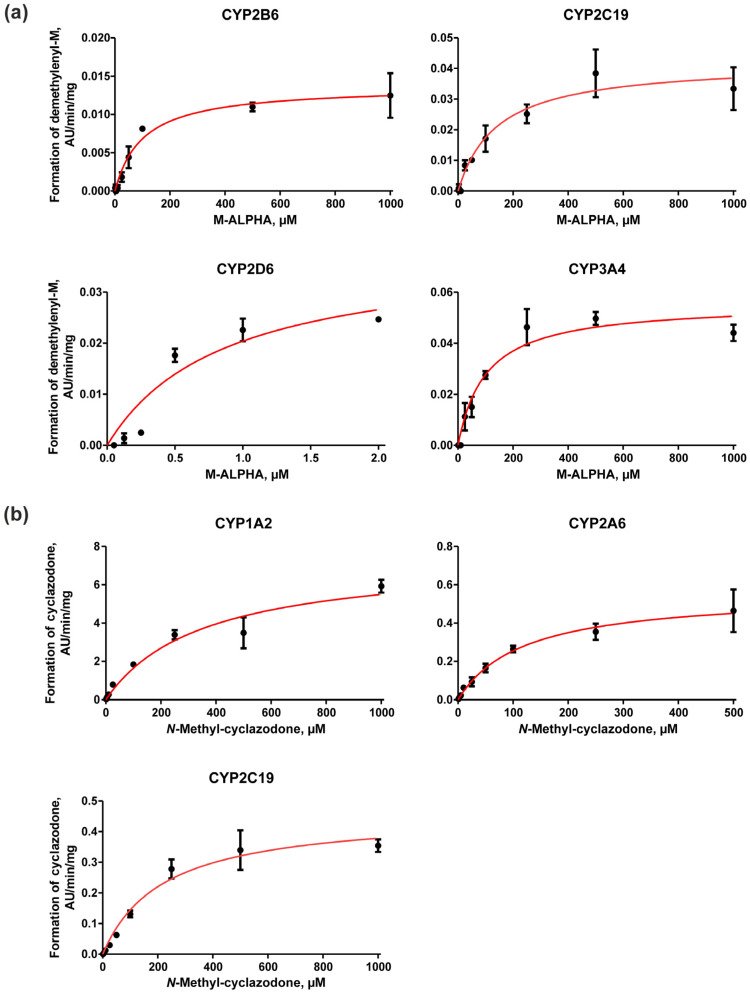
Michaelis–Menten plots for (**a**) demethylenation of M-ALPHA catalyzed by CYP2B6, CYP2C19, CYP2D6, and CYP3A4; and (**b**) formation of cyclazodone by CYP1A2, CYP2A6, and CYP2C19. Data points represent mean and ± standard error of the mean (SEM; *n* = 2).

Kinetic studies were performed based on the results of the screening of CYP-mediated metabolism. For M-ALPHA, the demethylenylation reaction was monitored; for *N*-methyl-cyclazodone, the formation of cyclazodone was monitored. The K_m_ and V_max_ values of both metabolic reactions for each CYP enzyme are listed in Table 1. All incubation profiles followed the expected classical hyperbolic Michaelis–Menten kinetics as shown in Figure 5, (a) M-ALPHA and (b) *N*-methyl-cyclazodone. 

Notably, CYP2D6 turned out to have the highest affinity towards demethylenation of M-ALPHA, as indicated by the low K_m_ value of 1.1 µM. Similar findings have also been reported by Meyer et al. for MDMA [29]. Turnover rates of the demethylenation reaction of M-ALPHA were comparably low across all tested CYP enzymes. In case of *N*-methyl-cyclazodone, K_m_ values were considerably higher than those observed for the demethylenation of M-ALPHA by CYP2D6, while the highest affinity for cyclazodone formation was observed with CYP2A6. Among all CYP enzymes investigated, CYP1A2 exhibited the highest turnover rate for cyclazodone formation. 

**Figure 6:**
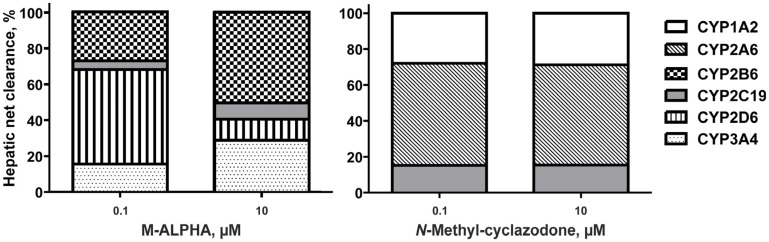
Calculated in vivo hepatic net clearances of contributing CYP enzymes on the demethyenylation of M-ALPHA (**right**) and cyclazodone formation (**left**) at concentration of 0.1 µM and 10 µM.

Based on these kinetic results, the in vivo hepatic net clearance for each compound was calculated at a low and a high concentration (0.1 and 10 µM), which is shown in Figure 6.

Demethylenylation of M-ALPHA was primarily mediated by CYP2D6 at low substrate concentrations, accounting for approximately 53% of the reaction. However, it becomes considerably less important with a high substrate concentration of about 12%, a trend that has also been reported for MDMA [29]. In contrast, CYP2B6 showed a reversed concentration–response pattern, with 27% of metabolism at low substrate concentrations attributed to this enzyme. Its relative contribution increased at higher concentrations, reaching 50%—a shift that was less pronounced for MDMA [29]. CYP3A4 and CYP2C19 started with comparatively low values of 5% and 15% and almost doubled at the high substrate concentration. This concentration-dependent shift suggests that CYP2D6 may be saturable, leading to compensatory metabolism primarily by CYP2B6 and to a lesser extent by CYP2C19 and CYP3A4 at higher substrate concentrations. Nevertheless, CYP2B6 and CYP2D6 remained the main contributors to the metabolism of M-ALPHA, accounting together for 80% at low concentration and 62% at high concentration.

Cyclazodone formation was predominantly mediated at the low substrate concentration by CYP2A6 (57%), followed by CYP1A2 (28%) and CYP2C19 (15%) with minor contributions. These proportions remained approximately constant across the two investigated concentrations, indicating that enzyme saturation is unlikely and that CYP2A6 also remains the dominant enzyme in *N*-methyl-cyclazodone metabolism at high concentrations. Co-administration with CYP2A6 inhibitors such as those discussed in the context of potential smoking cessation therapies or genetically decreased CYP2A6 activity may therefore increase the likelihood of toxic effects [32].

### 3.4. CYP Inhibition Potential of M-ALPHA

**Figure 7:**
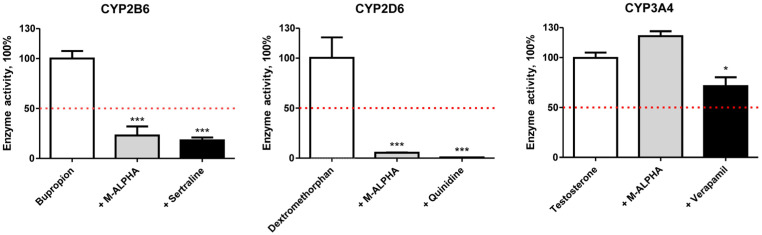
Inhibition potential of M-ALPHA determined for CYP2B6, CYP2D6, and CYP3A4. Negative controls (white bars) contained the respective CYP-specific substrate only, without any inhibitor, and were set to 100% enzyme activity. The red dotted line represents 50% enzyme activity. All other incubations additionally contained either 100 µM M-ALPHA (grey bars) or 20 µM of a CYP-specific inhibitor (sertraline for CYP2B6, quinidine for CYP2D6, and verapamil for CYP3A4; black bars). Each bar represents the mean of three incubations ± SEM. Statistical analysis was performed using one-way ANOVA followed by Dunnett’s post hoc test (*** *p* < 0.001, * *p* < 0.05).

MDMA and its derivative have been shown to possess inhibitory effects toward several CYP enzymes which could lead to potential interactions [33]. Effects of M-ALPHA on CYP2B6, CYP2D6, and CYP3A4 activity using individual CYP isoforms with their respective selective substrates bupropion (CYP2B6), dextromethorphan (CYP2D6), and testosterone (CYP3A4); and inhibitors sertraline (CYP2B6), quinidine (CYP2D6), and verapamil (CYP3A4) at concentrations specified by Dinger et al. are illustrated in Figure 7 [25].

CYP2D6 was most strongly affected by M-ALPHA with a reduction in enzymatic activity to 5% compared with less than 1% remaining activity after incubation with the model inhibitor quinidine. Activity of CYP2B6 was reduced by M-ALPHA to 23%, which was slightly less pronounced than the effect of the model inhibitor sertraline (18%). In contrast, CYP3A4 activity was not affected by M-ALPHA. With respect to CYP2D6 and CYP3A4, similar observations were reported for the phenethylamine analogue MDMA. However, no inhibition of CYP2B6 was observed for MDMA [33], which may result from structural differences compared to M-ALPHA that reduce binding affinity at the enzyme’s active site.

M-ALPHA appears to be a potent inhibitor of CYP2D6 and moderate inhibitor of CYP2B6. Similar to MDMA, CYP2D6 inhibition by M-ALPHA is expected to be mechanism-based but time-dependent [34]. Furthermore, as CYP2D6 and CY2B6 are the primary enzymes responsible for the metabolism of M-ALPHA and are inhibited strongly and moderately by M-ALPHA, respectively, these findings could have clinical relevance in polydrug use as described for MDMA, particularly when other CYP2D6 substrates are involved [35]. Nonetheless, these findings provide only an initial overview of M-ALPHA’s inhibitory potential, and a more comprehensive assessment would require determination of IC50 values.

### 3.5. Plasma Protein Binding

The extent of PPB often has a significant impact on its metabolism and excretion as well as drug–drug interactions. Results of the PPB study of M-ALPHA and *N*-methyl-cyclazodone are presented in Table 2.

A *f*_u_ value of 0.60 was calculated for M-ALPHA resulting in a PPB of 40%. Losacker et al. reported PPB values for (R)-MDMA and (S)-MDMA of 21.6% and 21.3%, respectively [36]. The nearly doubled PPB of M-ALPHA may be explained by its altered physicochemical properties, as reflected by a higher cLogP (2.3 versus 1.9 for MDMA). For *N*-methyl-cyclazodone, a *f*_u_ value of 0.64 was calculated which yielded in a PPB of 36%. This finding correlates with in vivo PPB data from two individual subjects receiving a 50 mg oral dose of pemoline [37]. Due to low-to-moderate PPB of both NPSs, significant drug–drug interactions are unlikely.

### 3.6. Limitations

We must acknowledge that our results are either based on in vitro models or on rats as an in vivo model, which limits extrapolation to potential human outcomes. For a comprehensive toxicokinetic profile including a reliable prediction of clearance, key in vivo pharmacological parameters such as plasma concentration–time data, AUC, C_max_, and plasma half-life must be determined. However, due to ethical concerns, human studies are usually not feasible for NPSs. Another relevant toxicokinetic aspect is the extent of absorption of the stimulants and their penetration of the blood–brain barrier, which was not part of this manuscript.

## 4. Conclusions

The present study characterized the toxicokinetics of the NPSs M-ALPHA and *N*-methyl-cyclazodone using a combination of in vitro and in vivo tools. Metabolism studies of M-ALPHA revealed metabolites comparable to those of its positional isomer MDMA. Both M-ALPHA and MDMA, as well as metabolites, share identical chemical formulas and highly similar HRMS/MS spectra. Together with only minor retention time shifts, the reliable differentiation of these regioisomers remains a challenge in forensic and clinical toxicology. Kinetic studies demonstrated that M-ALPHA metabolism is primarily mediated by CYP2B6 and CYP2D6. M-ALPHA was found to concurrently inhibit both isoforms, potentially leading to autoinhibition or an increased risk of toxic drug–drug interactions (DDIs) when co-ingested with other CYP2D6 or CYP2B6 substrates. In contrast, *N*-Methyl-cyclazodone was metabolized exclusively to cyclazodone, primarily mediated by CYP2A6. These findings suggest that the metabolic clearance of *N*-methyl-cyclazodone may be altered in individuals with CYP2A6 genetic polymorphisms or in the presence of potent CYP2A6 inhibitors. Detectability studies showed that a recreational intake should at least be detectable by HPLC-based SUSAs primarily using the described metabolites at targets.

## Figures and Tables

**Table 1:** Michaelis–Menten parameters for selected CYP enzymes of demethylenation of M-ALPHA and cyclazodone formation.

Metabolite ID	Enzyme	K_m, µM_	V_max, AU/min/mg_
**M-ALPHA**			
A1 Demethylenation	CYP2B6	101	0.01
	CYP2C19	141	0.04
	CYP2D6	1.1	0.04
	CYP3A4	101	0.06
***N-*** **Methyl-cyclazodone**			
B1 Cyclazodone	CYP1A2	352	7.4
	CYP2A6	119	0.6
	CYP2C19	219	0.5

**Table 2:** Unbound fraction (*f*_u_) and plasma protein binding (PPB) for M-ALPHA and *N*-methyl-cyclazodone.

Compound	*f*_u_	PPB, %
M-ALPHA	0.60	40 ± 4
*N*-Methyl-cyclazodone	0.64	36 ± 5

## Data Availability

The data that support the findings of this study are available from the corresponding author upon reasonable request.

## Supplementary Materials

The following supporting information can be downloaded at: https://www.mdpi.com/article/10.3390/metabo16050291/s1, Table S1: Identification of M-ALPHA and its metabolites sorted by increasing mass and retention time (Rt); Table S2: Identification of *N*-methyl-cyclazodone and its metabolite sorted by increasing mass and retention time (Rt).
