# Supplementary material for: Toxicokinetic Studies of the Two Stimulants M-ALPHA and N-Methyl-cyclazodone Using In Vitro and In Vivo Tools
Source: Metabolites. 2026 Apr 23;16(5):291. doi: 10.3390/metabo16050291 (PMC13208520; doi:10.3390/metabo16050291)
Supplement: Supplementary file 1 [file metabolites-16-00291-s001.zip › metabolites-4249622-supplementary.pdf]

Metabolites

Supporting Information

Toxicokinetic studies of the two stimulants M-ALPHA and *N*-methyl-cyclazodone using in vitro and in vivo tools

Tanja M. Gampfer, Samira Klaes, Niels Eckstein, Markus R. Meyer

Table S1 Identification of M-ALPHA and its metabolites sorted by increasing mass and retention time (Rt). For each metabolite its ID, metabolic reaction, measured precursor ion (PI) and the three most abundant fragment ions (FI), elemental composition, calculated exact mass, and mass error between calculated and measured mass are specified.

**Metabolite ID**	**Metabolic reaction**	**Measured masses of PI and characteristic FI**	**Elemental composition**	**Calculated exact mass [m/z]**	**Mass error [ppm]**	**Rt [min]**
M-ALPHA	-	PI at *m/z* 194.1178	$C_{11}H_{16}O_2N$	194.1175	1.09	3.9
		FI at *m/z* 163.0755	$C_{10}H_{11}O_2$	163.0764	0.79	
		FI at *m/z* 135.0443	$C_8H_7O_2$	135.0451	1.44	
		FI at *m/z* 133.0650	C_9H_9O	133.0647	1.47	
A1	Demethylenation	PI at *m/z* 182.1178	$C_{10}H_{16}O_2N$	182.1175	1.50	2.2
		FI at *m/z* 151.0756	$C_9H_{11}O_2$	151.0753	0.23	
		FI at *m/z* 123.0444	$C_7H_7O_2$	123.0440	2.63	
		FI at *m/z* 105.0704	C_8H_9	105.0698	5.02	
A2	Demethylenation + *O*-methylation	PI at *m/z* 196.1329	$C_{11}H_{18}O_2N$	196.1332	-1.30	3.1
		FI at *m/z* 154.0864	$C_8H_{12}O_2N$	154.0862	0.71	
		FI at *m/z* 137.0599	$C_8H_9O_2$	137.0597	1.12	
		FI at *m/z* 91.0548	C_7H_7	91.0542	6.32	
A3	Demethylenation + glucuronidation isomer 1	No PI detected	$C_{16}H_{24}O_8N$	358.1496	-	0.5
		FI at *m/z* 151.0755	$C_9H_{11}O_2$	151.0753	0.95	
		FI at *m/z* 123.0443	$C_7H_7O_2$	123.0440	1.82	
		FI at *m/z* 105.0703	C_8H_9	105.0698	4.43	
A4	Demethylenation +glucuronidation isomer 2	No PI detected	$C_{16}H_{24}O_8N$	358.1496	-	1.1
		FI at *m/z* 151.0755	$C_9H_{11}O_2$	151.0753	0.95	
		FI at *m/z* 123.0442	$C_7H_7O_2$	123.0451	1.64	
		FI at *m/z* 105.0703	C_8H_9	105.0698	4.29	

Table S2 Identification of *N*-methyl-cyclazodone and its metabolite sorted by increasing mass and retention time (Rt). For each metabolite its ID, metabolic reaction, measured precursor ion (PI) and the three most abundant fragment ions (FI), elemental composition, calculated exact mass, and mass error between calculated and measured mass are specified.

**Metabolite ID**	**Metabolic reaction**	**Measured masses of PI and characteristic FI**	**Elemental composition**	**Calculated exact mass [m/z]**	**Mass error [ppm]**	**Rt [min]**
N-Mehyl-cyclazodone	-	PI at *m/z* 231.1130	$C_{13}H_{15}O_2N_2$	231.1128	0.68	5.7
		FI at *m/z* 160.1122	$C_{11}H_{14}N$	160.1120	0.76	
		FI at *m/z* 132.0446	C_8H_6ON	132.0443	1.60	
		FI at *m/z* 113.0350	$C_4H_5O_2N_2$	113.0345	3.64	
B1	*N*-Demethylation	PI at *m/z* 217.0971	$C_{12}H_{13}O_2N_2$	217.0971	-0.35	5.0
		FI at *m/z* 146.0966	$C_{10}H_{12}N$	146.0964	0.90	
		FI at *m/z* 106.0656	C_7H_8N	106.0651	4.66	
		FI at *m/z* 91.0549	C_7H_7	91.0553	7.07	
